# Prevalence, antimicrobial susceptibility pattern, and associated factors of *Salmonella* and *Shigella* among food handlers in Adigrat University student’s cafeteria, northern Ethiopia, 2018

**DOI:** 10.1186/s40794-020-00119-x

**Published:** 2020-09-11

**Authors:** Haftom Legese, Tsega Kahsay, Aderajew Gebrewahd, Brhane Berhe, Berhane Fseha, Senait Tadesse, Guesh Gebremariam, Hadush Negash, Fitsum Mardu, Kebede Tesfay, Gebre Adhanom

**Affiliations:** 1 Department of Medical Laboratory, College of Medicine and Health Science, Adigrat University, Adigrat, Ethiopia; 2 Department of public health, College of Medicine and Health Science, Adigrat University, Adigrat, Ethiopia; 3 Department of Medical Laboratory, College of Medicine and Health Science, Bahr dar University, Bahr dar, Ethiopia

**Keywords:** Antimicrobial susceptibility, Food handlers, *Salmonella*, *Shigella*, Ethiopia

## Abstract

**Background:**

Food handlers play a significant role in the transmission of foodborne infections. *Salmonella* and *Shigella* are the most common foodborne pathogens and their infections are a major public health problem globally. Thus, this study aimed to determine the prevalence, antimicrobial susceptibility patterns, and associated factors of *Salmonella* and *Shigella* colonization among food handlers.

**Methodology:**

A cross-sectional study was conducted from March to August 2018 at Adigrat University student cafeteria, Northern Ethiopia. Data on socio-demographic and associated factors were collected using a structured questionnaire. Fresh stool samples were collected from 301 food handlers and transported to Adigrat University Microbiology Laboratory. Bacterial isolation and antimicrobial susceptibility test were performed using standard bacteriological methods. Data analysis was performed using SPSS version 22 and *P* < 0.05 where a corresponding 95% confidence interval was considered statistically significant.

**Results:**

A total of 301 food handlers were included in this study. The majority of study participants were females 265 (88.0%). About 22 (7.3%) and 11 (3.7%) of food handlers were found to be positive for *Salmonella* and *Shigella* respectively. Hand washing after using a bathroom with water only, no hand washing after using the bathroom, no hand washing after touching dirty materials, no hand washing before food handling, and untrimmed fingernails were significant associated factors identified. None of the *Salmonella* and *Shigella* isolates were sensitive to ampicillin, yet low resistance against chloramphenicol, ceftriaxone, and ciprofloxacin was found.

**Conclusion:**

The present study revealed that the prevalence of *Salmonella* and *Shigella* among food handlers was 22 (7.3%) and 11 (3.7%) respectively. Such colonized food handlers can contaminate food, and drinks and could serve as a source of infection to consumers. This indicates that there is a need for strengthened infection control measures to prevent *Salmonella* and *Shigella* transmission in the students’ cafeteria.

## Background

Foodborne diseases are a major public health problem globally. The severity is higher among developing countries due to low hygienic food handling practices, lack of environmental sanitation, and poor access to safe drinking water [1]. In developing countries, approximately 70% of cases of diarrheal diseases are associated with the consumption of contaminated food [2].

*Salmonella* remains a major cause of foodborne infection in humans [3], which leads to approximately 93 million infections every year [4, 5]. The World Health Organization (WHO) estimates that there are around 16 million new cases and 600,000 deaths due to typhoid fever each year worldwide [6]. It causes bacterial bloodstream infections with a fatality rate of 20–25% [7]. The widespread nature of salmonellosis increases antibiotic resistance which in turn increases the treatment cost, hospitalization, morbidity, and mortality [8].

These bacteria are transmitted directly and indirectly through contaminated objects such as food, water, nails, and fingers. This indicates those microorganisms can be spread by fecal-oral human-to-human transmission [9, 10]. Compared to other parts of the hand, fingernails harbor the most microorganisms and are difficult to clean.

*Shigella* continues to play a major role in the etiology of inflammatory diarrhea and dysentery in food handlers [11]. The annual incidence of *Shigella* is estimated to be 164.7 million people, with 69% of all deaths attributable to shigellosis worldwide [12, 13]. The highest prevalence of shigellosis is observed in tropical and subtropical parts of the world [14].

*Salmonella* and *Shigella* are a significant cause of severe post-diarrheal complications such as reactive arthritis, sepsis, Reiter’s syndrome, myocarditis, inflammatory bowel diseases, irritable bowel syndrome, and peritonitis [8, 15, 16]. The emergence of antimicrobial-resistant *Salmonella* and *Shigella* becomes a significant threat to deliver reliable therapies [17, 18].

In Ethiopia, it is difficult to estimate the severity of salmonellosis and shigellosis as well as their antibiotic resistance due to the limited scope of studies, lack of coordinated epidemiological surveillance system, poor reporting system, and limited availability of culture facilities [19].

Determining the prevalence and antimicrobial susceptibility pattern of *Salmonella* and *Shigella* is very important for the proper selection of antimicrobial agents to control the spread of infection. However, in the study area, there was a scarcity of data on the carriage of *Salmonella* and *Shigella* among food handlers. Therefore, this study aims to assess the prevalence, antimicrobial susceptibility patterns, and associated factors of *Salmonella* and *Shigella* among food handlers in Adigrat University, Tigrai, Northern Ethiopia.

## Materials and methods

### Study design, area and period

A cross-sectional study was conducted among food handlers who participated in food preparation, dispatch and storage at the Adigrat University student cafeteria from March to August 2018. The annual rainfall ranges from 400 to 600 mm and the minimum and maximum temperature range from 6 to 21.8 °C. Currently, the university enrolls more than 15,000 students dining in the student cafeteria. There are six cafeterias, and a total of 700 food handlers are working in the student cafeteria (Adigrat University human resource management and registrar office). Few food handlers in the university received food handling certification and graduated in food handling, preparation, and serving programs. Although there was a periodic medical checkup in the university, it was not consistent. In contrast to the university food handlers, food handlers employed in serving the public and tourists are well-trained and graduate in food preparation and handling practice from college or universities.

### Sample size determination and sampling technique

#### Sample size determination

The sample size was determined by using a single population proportion formula.

$$ \mathrm{n}=\frac{{\left(\mathrm{Z}\upalpha /2\right)}^2\mathrm{P}\ \left(1-\mathrm{P}\right)}{{\mathrm{d}}^2} $$

The sample size was determined based on the prevalence of *Salmonella* among university food handlers done by Mama and Alemu (2016) at Arba Minch University, South Ethiopia (6.9%) [14]. Then with a margin of error (5%), (d = 0.03) and 95% level of confidence (z = 1.96), the sample size was calculated as follows:

$$ \mathrm{n}=\frac{(1.96)^2\ast 0.069(0.931)}{(0.03)^2}=274,\mathrm{with}\ 10\%\mathrm{non}\ \mathrm{response}\ \mathrm{rate}=301 $$

Therefore, a total of 301 food handlers were included in the study from all university cafeterias. A simple random sampling technique was employed. The lottery method was used to select the study subjects after a complete list of food handlers was obtained from a roster of cafeteria staff at Adigrat University.

### Eligibility criteria

#### Inclusion criteria

Food handlers working in Adigrat University student cafeterias were included in the study.

#### Exclusion criteria

Food handlers who have taken antibiotics and/or antihelminthics within one week and those with clinical signs of typhoid fever such as cough, a high temperature which reaches up to 39 to 40C, headache, general aches and pains were excluded from the study.

### Data collection and sample processing

#### Socio-demographic and specimen collection, handling and transportation

A structured questionnaire was used to collect the data regarding socio-demographic and associated factors. Questionnaires were checked for accuracy and completeness. After proper instruction, about 2 g of fresh stool specimens were collected from food handlers with a labeled wide-mouthed plastic container and a clean wooden applicator stick. Specimens were immediately transported to the laboratory using an icebox.

#### Isolation and identification

The stool specimen was collected and transported to Adigrat University Medical Microbiology Laboratory within one hour of collection. Stool specimens were immediately inoculated in Selenite F enrichment broth and incubated at 37 °C for 24 h, and then subculture onto selective media of xylose-lysine desoxycholate agar (XLD) and Hektoen enteric medium agar at 37 °C for 18–24 h. The isolated colonies were differentiated and identified based on Gram stain, colonial morphology and pigmentation, hemolysis on blood agar, catalase test, oxidase test, carbohydrate fermentation, H_2_S production, motility, indole formation, urease production, and citrate utilization. Cultures were incubated for 24 to 48 h at 37 °C. Then colonies producing an alkaline slant with acid butt and hydrogen sulfide production on Triple Sugar Iron Agar, positive for lysine, negative for urea hydrolysis, negative for indole test, positive for citrate utilization and motility test were considered to be *Salmonella*. Colonies which were urease negative, indole positive/negative, produced a pink-red slope and yellow butt with no blackening on Triple Sugar Iron agar, lysine decarboxylase negative and citrate negative were identified as *Shigella* species. Finally, all of the confirmed *Salmonella* and *Shigella* isolates were examined for antimicrobial susceptibility.

#### Antimicrobial susceptibility tests

Antimicrobial susceptibility testing was performed using the modified Kirby-Bauer disc diffusion method according to Clinical and Laboratory Standards Institute (CLSI) guidelines, 2016 [20]. Using a sterile wire loop, 3–5 well-isolated colonies of the test organism was emulsified into a tube of 3–4 ml sterile physiological saline to get bacterial inoculums equivalent to 0.5 McFarland turbidity standards. Then the standardized suspension (test organisms) were uniformly swabbed within 15 min using a sterile cotton swab into Muller-Hinton agar and allowed to dry. After that, the antibiotic discs were placed manually on the medium and incubated at 37 °C for about 18 h and the zones of inhibition were measured using a caliper. The interpretation of the results was made based on the CLSI criteria as sensitive, intermediate and resistant [20]. The following antimicrobials were prioritized by considering local prescription; gentamicin (10 μg), ampicillin (30 μg), amoxicillin (30 μg), ciprofloxacin (5 μg), clarithromycin (30 μg), chloramphenicol (30 μg), cotrimoxazole (25 μg), amoxicillin-clavulanic acid (30 μg), and ceftriaxone (30 μg) [20].

#### Data quality assurance

Data quality was ensured at various activities of the study by following a prepared standard operating procedure (SOP). Questionnaires were prepared in a clear and precise way and translated into the local language and back-translated to English to ensure the consistency of the questionnaires. The pretest was done on 5% of food handlers and modifications were made accordingly. To ensure general safety, universal bio-safety precautions were followed. American Type Culture Collection (ATCC) strains *P.aeruginosa* (ATCC 27853), *E. coli* (ATCC 25922) were used as control strains for the culture and antimicrobial susceptibility testing.

### Statistical analysis

After the collection of socio-demographic characteristics, associated factors and laboratory data using a structured questionnaire and laboratory report format, data were edited, cleaned, entered and analyzed using Statistical Package for Social Sciences (SPSS) version 22. Descriptive statistics, bivariate, and multivariate logistic regressions were performed. Bivariate logistic regression was employed to determine the association between the outcome variable and each independent variable. A binary logistic regression analysis was used to calculate the odds ratios (OR); crude odds ratio (COR) and adjusted odds ratio (AOR) to ascertain the degree of association between dependent and independent variables. All variables with *p*-value 0.20 in the bivariate logistic regression were transferred to multivariate logistic regression analysis to compute AOR. The regression model was first examined by the goodness of fit test using the Hosmer-Lemeshow test to determine whether the model is adequately fitted to the study. In this study, multi-collinearity among independent variables was detected using the standard errors for regression coefficients. Finally, variables with a p-value < 0.05 with a corresponding 95% confidence interval (CI) were considered as statistically significant.

### Operational definition

Dirty materials: Dirty materials are soiled objects, unclean or impure.

Fingernail status: Status of the nail was either trimmed or untrimmed fingernails that serve as a vehicle for the transmission of food contaminating pathogens.

## Results

### Socio-demographic characteristics

A total of 301 food handlers were included in the study. Out of the total respondents, 265 (88.0%) were females. The age of study participants ranged from 19 to 38 years (23.51 ± 3.186 years). The majority of 241 (80.1%) of the participants were between the ages of 21 and 30 years. One hundred fifty-six (51.8%) were enrolled in secondary school with an average of 3.7 years of work experience in the cafeteria. Out of the total study participants, 37 (12.3%) were certified for training in food handling and 265 (88.0%) had previously undergone a medical checkup stool microscopy examination **(**Table 1**)**.

**Table 1:** Socio-demographic characteristics of food handlers in Adigrat University student cafeteria, Tigrai, North Ethiopia March to August 2018 (*N* = 301)

Demographic characteristics	Characteristics	Frequency	Percent
**Sex**	Male	37	12.3
	Female	264	87.7
**Age**	≤ 20	54	17.9
	21–40	247	80.1
**Marital status**	Single	127	42.2
	Married	174	57.8
**Educational level**	Secondary school	187	62.12
	Higher than secondary school	114	37.9
**Years of service**	≤1	36	12.0
	1–2	42	14.0
	> 2	223	74.1
**Certified in food preparation and handling**	No	264	87.7
	Yes	37	12.3
**Medical check-up**	No	36	12.0
	Yes	265	88.0

### Prevalence and associated factors of *Salmonella* and *Shigella* carriers

The prevalence of *Salmonella* and *Shigella* in this study was 22 (7.3%) and 11 (3.7%) respectively. In the current study, 13 independent variables were considered during the analysis of associated factors for *Salmonella* and *Shigella* carriers **(**Table 2**)**. Accordingly in the multivariate analysis, hand washing after using the bathroom with water only (AOR = 23.24, 95% CI: 2.13–254.17, *P* < 0.01), no handwashing after using the bathroom (AOR = 2.25, 95% CI: 5.11–77.34, *P* < 0.001), no hands washing after touching dirty materials (AOR = 37.19, 95% CI: 5.66–244.45, P < 0.001), no handwashing before food handling (AOR = 33.1, 95% CI: 4.96–220.52, P < 0.001), untrimmed fingernail (AOR = 13.97, 95% CI: 3.40–57.36, P < 0.001) were significant factors associated with *Salmonella* and *Shigella* carriers. However, no significant association was found between years of service, medical check-up, workers blowing their noses, touching food with bare hands, and certification in food preparation and handling, with *Salmonella* and *Shigella* colonization in this study **(**Table 3**)**.

**Table 2:** Bivariate logistic regression analysis of factors associated with *Salmonella* and *Shigella* infections among food handler’s working at Adigrat University Students’ Cafeteria, Tigrai, Northern Ethiopia, March to August 2018 (N = 301)

Variables		Growth of ***Salmonella*** and ***Shigella***		COR (95% CI)	***P***-value
		Positive N (%)	Negative N (%)		
**Sex**	Male	1 (2.7)	36 (97.3)	0.22 (0.03–1.58)	0.20
	Female	32 (12.1)	232 (87.9)	**1**	
**Age**	≤ 20	2 (3.7)	52 (96.3)	0.3 (0.07–1.2)	0.081
	21–40	31 (12.55)	216 (87.45)	**1**	
**Marital status**	Single	16 (12.6)	111 (87.4)	1.29(.68–2.45)	0.341
	Married	17 (9.78)	157 (90.23)	**1**	
**Educational status** **Hand washing after using the bathroom**	Lower than Secondary school	16 (8.56)	171 (91.44)		0.507
	Higher than secondary school	17 (14.9)	97 (85.1)	1.74 (0.92–3.31)	0.862
	Yes with water and soap	1 (0.9)	112 (99.1)	**1**	
	Yes only with water	17 (13.6)	108 (86.4)	17.630 (2.306–134.7)	0.006
	No	15 (23.8)	48 (76.2)	35.000 (4.495–272.49)	0.001
**Hand washing after touching dirty materials**	Yes with water and soap	5 (3.4)	141 (96.6)	**1**	
	Yes only with water	14 (11.0)	113 (89.0)	3.494 (1.222–9.991)	0.020
	No	14 (50.0)	14 (50.0)	28.200 (8.845–89.906)	0.001
**Handwashing before food handling**	Yes with water and soap	2 (1.8)	112 (98.2)	1	
	Yes only with water	13 (9.2)	129 (90.8)	5.643 (1.247–25.548)	0.025
	No	18 (40.0)	27 (60.0)	37.333 (8.164–170.714)	0.001
**Fingernail status**	Trimmed	4 (2.2)	174 (97.8)	**1**	
	Untrimmed	29 (23.6)	94 (76.4)	10.490 (3.780–29.09)	0.001
**After blowing nose**	Yes with water and soap	7 (8.0)	81 (92.0)	**1**	
	Yes only with water	11 (11.7)	83 (88.3)	0.919 (0.401–2.107)	0.842
	No	15 (12.6)	104 (87.4)	0.599 (0.233–1.538)	0.287
**Touch food with bare hands**	No	16 (14.0)	98 (86.0)	**1**	
	Yes	17 (9.1)	170 (90.9)	1.597 (0.772–3.302)	0.207
**Years of service**	≤1	9 (25.0)	27 (75.0)	3.72 (1.76–78.50)	0.001
	1–2	9 (21.4)	33 (78.6)	0.818 (0.285–2.349)	0.709
	> 2	15 (6.7)	208 (93.3)	**1**	
**Certified in food preparation and handling**	No	29 (11.5)	224 (88.5)	**1**	
	Yes	4 (8.3)	44 (91.7)	0.702 (0.235–2.097)	0.527
**Medical check-up**	No	10 (18.5)	44 (81.5)	**1**	
	Yes	23 (9.3)	224 (90.7)	1.99 (1.01–3.93)	0.04

Key: ^a^ (COR = Crude odds ratio); ^b^ (CI=Confidence interval);1(referent)

**Table 3:** Multivariate logistic regression analysis of factors associated with *Salmonella* and *Shigella* isolates among food handler’s working at Adigrat University Students’ Cafeteria, Tigrai, Northern Ethiopia, March to August 2018 (N = 301)

Variables	Growth of ***Salmonella*** and ***Shigella***		COR^**a**^ (95% CI^**b**^)	P-value	AOR^**c**^ (95%CI)	P-value
	Positive N (%)	Negative N (%)				
**Hand washing using after using the bathroom**						
Yes with water and soap	1 (0.9)	112 (99.1)	**1**		**1**	
Yes only with water	17 (13.6)	108 (86.4)	17.630 (2.306,134.7)	0.006	23.239 (2.125–254.17)	0.010*
No	15 (23.8)	48 (76.2)	35.000 (4.495,272.49)	0.001	62.917 (5.11–77.34)	0.001*
**Hand washing after touching dirty materials**						
Yes with water and soap	5 (3.4)	141 (96.6)	**1**		**1**	
Yes only with water	14 (11.0)	113 (89.0)	3.494 (1.222, 9.991)	0.020	1.089 (0.187–6.345)	0.924
No	14 (50.0)	14 (50.0)	28.200 (8.845, 89.906)	0.000	37.189 (5.658–244.45)	0.001*
**Hand washing before food handling**						
Yes with water and soap	2 (1.8)	112 (98.2)	**1**		**1**	
Yes only with water	13 (9.2)	129 (90.8)	5.643 (1.247, 25.548)	0.025	5.972 (0.899–39.677)	0.064
No	18 (40.0)	27 (60.0)	37.333 (8.164, 170.714)	0.000	33.065 (4.958–220.52)	0.001*
**Finger nail status**						
Trimmed	4 (2.2)	174 (97.8)	**1**		**1**	
Untrimmed	29 (23.6)	94 (76.4)	10.490 (3.780, 29.09)	0.000	13.973 (3.404–57.362)	0.001*

Key: ^a^ (COR = Crude odds ratio); ^b^ (CI=Confidence interval); ^c^ (AOR = Adjusted odds ratio); 1(referent)

### Antimicrobial susceptibility patterns of *Salmonella* and *Shigella* isolates

Antimicrobial susceptibility patterns were performed for 22 *Salmonella* and 11 *Shigella* isolates against 9 antimicrobial agents. All *Salmonella* and *Shigella* isolates were resistant to ampicillin. 22(100%) *Salmonella* and 10(90.90%) *Shigella* isolates were resistant to gentamicin; and 21(95.50% *Salmonella* and 11(100%) *Shigella* isolates were resistant to amoxicillin. However, all *Salmonella* and *Shigella* isolates were susceptible to ciprofloxacin. Additionally, low resistance was observed for ceftriaxone, chloramphenicol and cotrimoxazole for *Salmonella* and *Shigella*. None of the isolates showed intermediate resistance **(**Table 4**)**. Multidrug resistance in this study is defined as resistance to at least three classes of antimicrobial agents and out of the thirty-three isolates 12 (54.5%) *Salmonella* and 10(90.90%) *Shigella* species were multidrug- resistant isolates **(**Table 5**)**.

**Table 4:** Antimicrobial susceptibility patterns of *Salmonella* and *Shigella* isolated from food handlers Of Adigrat University Students’ Cafeteria, Tigrai, Northern Ethiopia, March to August 2018 (*N* = 33)

Isolates(n)	Sensitivity pattern n(%)	Antimicrobial agents N (%)								
		GM	AML	AMP	CIP	CLR	TS	AMC	CHL	CRO
***Salmonella*** **n(22)**	S	22 (100)	1 (4.5)	0 (0.00)	22 (100)	18 (81.8)	20 (90.9)	13 (59.1)	22 (100)	22 (100)
	R	0 (0.00)	21 (95.5)	22 (100)	(0.00)	4 (18.2)	2 (9.1)	9 (40.9)	0 (0.00)	0 (0.00)
***Shigella*** **n (11)**	S	10 (90.9)	0 (0.00)	0 (0.00)	11 (100)	2 (18.2)	10 (90.9)	6 (54.5)	9 (81.2)	10 (90.9)
	R	1 (9.1)	11 (100)	11 (100)	0 (0.00)	9 (81.2)	1 (9.1)	5 (45.5)	2 (18.2)	1 (9.1)

Key: S = Sensitive R = Resistant, GM Gentamicin, AML Amoxicillin, AMP Ampicillin, CIP=Ciprofloxacin, CLR Clarithromycin, TS Cotrimoxazole, AMC Amoxicillin-clavulanic acid CHL Chloramphenicol, CRO Ceftriaxone

**Table 5:** Multidrug-resistant of *Salmonella* and *Shigella* isolated from food handler’s working at Adigrat University Students’ Cafeteria, Tigrai, Northern Ethiopia, March to August 2018 N = 33

	Antimicrobials	Resistant isolates no. (%)	
		***Salmonella***	***Shigella***
**For three**	AML,AMP,CLR	2 (16.67)	4 (40)
	AML,AMP,CHL	–	1 (10)
	AML,CLR,AMC	7 (58.34)	–
	AMP,CLR,AMC	1 (8.33)	–
	AML,AMP,TS	1 (8.33)	–
**FOR FOUR**	AML,AMP,CLR,AMC	1 (8.33)	3 (30)
**FOR FIVE**	AML,AMP,CLR,AMC,CHL	–	1 (10)
**FOR SIX**	GM,AML,AMP,CLR,TS,AMC	–	1 (10)
		12 (100)	10 (100)

Key: GM Gentamicin, AML Amoxicillin, AMP Ampicillin, CIP=Ciprofloxacin, CLR Clarithromycin, TS Cotrimoxazole, AMC Amoxicillin-clavulanic acid CHL Chloramphenicol, CRO, Ceftriaxone

MDR multidrug resistant; MDR definition for *Salmonella* and *Shigella* percent is computed from total number of *Salmonella* and *Shigella*

## Discussion

In this study, the prevalence of *Salmonella* among food handlers was 22 (7.3%). This was similar to studies carried out in Southern Ethiopia, Arba Minch University (6.9%) [14], and Nigeria, Abeokuta (5.5%) [21]. However, it was higher than the studies reported from Ethiopia, Addis Ababa (3.5%) [22], Bahir Dar (1.6%) [23], and Gondar (3.1%) [24]. On the other hand, our result was lower than the studies reported from Ethiopia, Addis Ababa (10.5%) [25], and Nigeria (42.3%) [26]. The variation might be attributed to poor personal hygiene and environmental sanitation differences among the study areas.

The prevalence of *Shigella* (3.7%) in our study is consistent with studies done in Arba Minch University, Southern Ethiopia (3%) [14]; Addis Ababa, Ethiopia (4.5%) [25], and Gondar Ethiopia (3.1%) [26]. However, our finding was lower than a study conducted in Nigeria (15.5%) [27]. These might be due to the differences in inconsistent training on food preparation and handling and hygiene practices of the food handlers.

In the present study, not practicing handwashing after using the bathroom among food handlers was significantly associated with *Salmonella* and *Shigella* carriers. Food handlers who hadn’t washed their hands after using the bathroom were more likely to be colonized with *Salmonella* and *Shigella* compared to those who washed with water and soap after using the bathroom. This finding was similar to a study conducted in Mekelle, Ethiopia [28], Gondar, Ethiopia [29], and Bahir Dar, Ethiopia [30]. The acquisition of *Salmonella* and *Shigella* is due to poor sanitary conditions, poor toilet facilities, and scarcity of availability of facilities used for handwashing practice. The majority of food handlers of the university reported that they washed their hands with only water and some of them did not wash their hands at all after using the bathroom.

Our findings also revealed that there is a statistically significant difference in handwashing after touching dirty materials among food handlers with *Salmonella* and *Shigella* carriers. Food handlers who did not wash their hands after touching dirty materials are twenty-eight fold more likely to be colonized with *Salmonella* and *Shigella* than those who washed with water and soap after touching dirty materials. This finding is consistent with a study conducted in Ethiopia, Bahir Dar [23]. This might be due to the absence of handwashing facilities within proximity of the food handlers’ workplace.

Our study showed that food handlers who washed their hand with soap and water before touching food were less likely to be colonized with *Salmonella* and *Shigella* than food handlers who did not wash their hand with soap and water before food preparation. This is in line with the finding of a similar study reported fromYebu Town Ethiopia [31]. In this study, the majority offood handlers practiced handwashing before handling food. However, a very large proportion (47.1%) washed their hands only with water. There are food handlers who apply some hygiene practice, though many of them do not use soap nor do they appreciate or understand the need for handwashing [32].

Furthermore, in this finding, untrimmed fingernails were significantly associated with *Salmonella* and *Shigella* colonization among food handlers. This study is similar to studies conducted in-Yebu Town, Ethiopia [31], and Arba Minch, Ethiopia [14]. This result might be due to the lifestyle of food handlers. Examination of fingernail contents of food handlers for *Salmonella* or *Shigella* is one way of indicating a source of possible food contamination [31]. However, the current study did not assess the *Salmonella* and *Shigella* carriage of fingernail contents.

*Salmonella* and *Shigella* carriers who are preparing and handling food daily can act assources of infection to the university community via the food chain. Therefore, regular training, medical check-up programs and accessibility of personal hygiene guidelines with intensive health education could be important to prevent and control the carriage.

Antimicrobial susceptibility pattern data showed that ciprofloxacin, ceftriaxone, gentamicin, chloramphenicol, and cotrimoxazole were effective against the *Shigella* isolates. Our finding was comparable with studies reported from Ethiopia, Haramaya University on ceftriaxone (16.7%) [33], Jimma on gentamicin (1.3%) [34], and Harar on gentamicin (3.6%) [35]. Whereas our result showed lower resistance patterns compared to the studies conducted in Ethiopia, Addis Ababa on gentamicin (75.6%) [36], and Gondar on ciprofloxacin (8.9%) and cotrimoxazole (73.4%) [37, 38]. This increase of resistance from those reports indicates that there are differences in the geographical area, study period and study design. Increased resistance was observed in our finding which is in line with a study reported from Harar on ampicillin (100%) [35], and Arba Minch on amoxicillin (100%) [14].

In the current study, isolates of *Salmonella* species were sensitive to gentamicin, ciprofloxacin, chloramphenicol, ceftriaxone, cotrimoxazole, and clarithromycin. This is consistent with reports from Gondar University, Ethiopia [24, 25, 38]. Increased resistance was observed in our findings for amoxicillin-clavulanic, amoxicillin, and ampicillin which were supported by studies reported from Ethiopia in Arba Minch, Jimma, and Bahir Dar [12, 14, 23, 35]. This might be due to misuse or inappropriate use of antibiotics and the use of clinical diagnosis for treatment by physicians may lead to the emergence of drug-resistant bacterial strains and replacement of sensitive strains by resistant strains.

The prevalence of multidrug resistance towards *Salmonella* and *Shigella* were observed. Out of all the isolates, 54.54% *Salmonella* and 90.9% *Shigella* species were resistant to at least three antimicrobials. One isolate of *Shigella* was resistant to six classes of antimicrobial agents. This study is supported by a study conducted in Ethiopia in Butajira [25], Addis Ababa [22], Haramaya University [33], and Gondar [24]. This increased multidrug resistance might be due to genetic variation by mutations, irrational use of antimicrobials, and less hygienic practice of the food handlers. *Salmonella* and *Shigella* species are becoming resistant to most antimicrobials, indicating that there might be easy availability, irrational use of common antimicrobials from different governmental and private pharmacies.

### Limitation

Fingernail content examination could not be identified. This may be supportive to know whether the contamination is due to poor fingernail hygiene or poor food handling practices. Despite this limitation, the methods used to isolate and characterize the antimicrobial susceptibility pattern of *Salmonella* and *Shigella* spp. are comprehensive. In the current study, because of the self-reported nature of the study, recall bias was a limitation but it is not reflected in the findings. We also do not know what biases have the greatest impact on self-reports. In addition to that, even though this study used a random sampling technique to select study participants, it was facility-based. The Hosmer-Lemeshow test is used for overall calibration error but not used for a particular lack of fitness, so it does not properly take overfitting into account. Therefore generalizability might be hardly achieved. Additionally, since there is variation in seasons, geography, and in the definition of antimicrobial resistance guidelines among different studies and across regions we couldn’t infer the external validity.

## Conclusion

The overall prevalence of *Salmonella* and *Shigella* in the study area was found to be 7.3 and 3.7% respectively. The *Salmonella* and *Shigella* carriage was significantly associated with not washing hands after touching dirty materials, not washing hands after using the bathroom, and untrimmed fingernails. No resistance against chloramphenicol, ceftriaxone, and ciprofloxacin was identified*.* The majority of *Salmonella* and *Shigella* were multidrug-resistant. To improve food handling safety within the university, regular medical checkups, increased handwashing, and environmental sanitation should be practiced. Consistent training on food preparation and handling for the food handlers of Adigrat University is very important to prevent the risk of infection for the university community having close contact with those carriers. Additionally, this study suggested that physicians should prescribe based on the laboratory result. Drug dispensing by different governmental and private pharmacies should be according to the prescription of the physicians.

## Data Availability

All data collected and analyzed during this study were included in the manuscript. But if the full paper is needed, it will be shared upon request by the editor from the corresponding author.

## Abbreviations

ATCC: American Type Culture Collection
CI: confidence interval
CLSI: Clinical and Laboratory Standards Institute
MDR: Multi-drug resistant Multi-Drug Resistant
SOPs: Standard Operating Procedures
USA: United States of America
WHO: World health organization
